# Supplementary material for: Dynamic antagonism between key repressive pathways maintains the placental epigenome
Source: Nat Cell Biol. 2023 Apr 6;25(4):579–91. doi: 10.1038/s41556-023-01114-y (PMC10104784; doi:10.1038/s41556-023-01114-y)
Supplement: Supplementary file 1 — Supplementary Fig. 1. [file 41556_2023_1114_MOESM1_ESM.pdf]

nature cell biology

Article https://doi.org/10.1038/s41556-023-01114-y

Dynamic antagonism between key repressive pathways maintains the placental epigenome

In the format provided by the authors and unedited

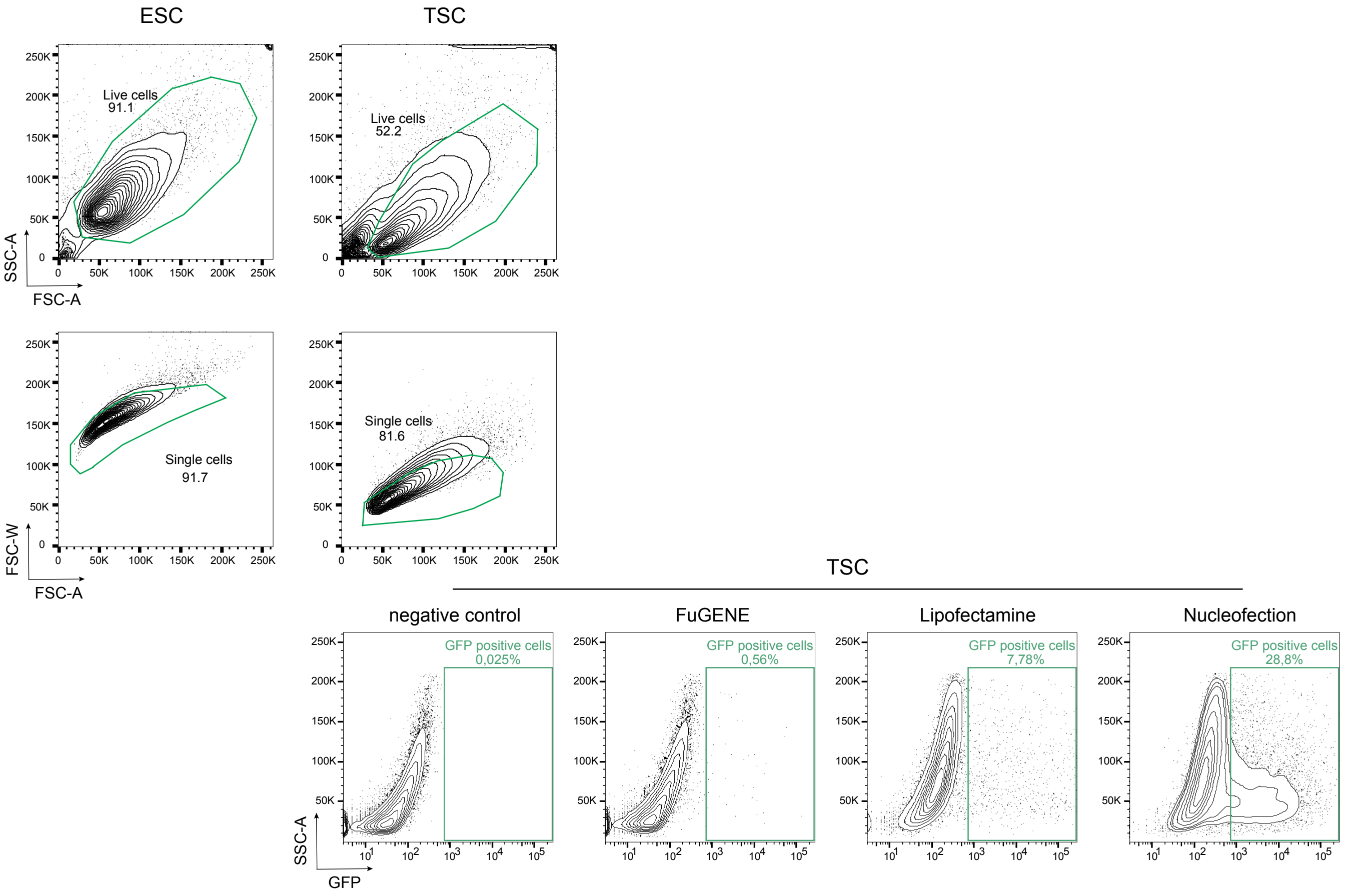

Supplementary Figure 1: Gating strategy for sorting embryonic and trophoblast stem cells (ESC, TSC). Top panel: FSC-A/SSC-A was used to determine live cells, followed by gating single cells using FSC-A/FSC-W. This gating was used for the ESCs and TSCs single cell expansion experiment. Cells were sorted onto a 96 well plate (1 cell/well). Bottom panel: For the TSC knockout lines, first, cells were gated according to the top panel (live and single cell), followed by sort for GFP positive cells. TSC wildtype cells were transfected with the corresponding sgRNA/Cas9 plasmid expressing GFP. TSC wildtype cells were used as negative control to set the GFP+ gate.
