# Supplementary material for: Dynamic antagonism between key repressive pathways maintains the placental epigenome
Source: Nat Cell Biol. 2023 Apr 6;25(4):579–91. doi: 10.1038/s41556-023-01114-y (PMC10104784; doi:10.1038/s41556-023-01114-y)
Supplement: Supplementary file 2 — Reporting Summary [file 41556_2023_1114_MOESM2_ESM.pdf]

nature research

Corresponding author(s): Alexander Meissner, Zachary D. Smith

Last updated by author(s): Dec 30, 2022

# Reporting Summary

Nature Research wishes to improve the reproducibility of the work that we publish. This form provides structure for consistency and transparency in reporting. For further information on Nature Research policies, see our Editorial Policies and the Editorial Policy Checklist.

## Statistics

For all statistical analyses, confirm that the following items are present in the figure legend, table legend, main text, or Methods section.

| n/a | Confirmed | |
|---|---|---|
| ☐ | ☒ | The exact sample size (*n*) for each experimental group/condition, given as a discrete number and unit of measurement |
| ☐ | ☒ | A statement on whether measurements were taken from distinct samples or whether the same sample was measured repeatedly |
| ☐ | ☒ | The statistical test(s) used AND whether they are one- or two-sided<br>*Only common tests should be described solely by name; describe more complex techniques in the Methods section.* |
| ☐ | ☒ | A description of all covariates tested |
| ☐ | ☒ | A description of any assumptions or corrections, such as tests of normality and adjustment for multiple comparisons |
| ☐ | ☒ | A full description of the statistical parameters including central tendency (e.g. means) or other basic estimates (e.g. regression coefficient) AND variation (e.g. standard deviation) or associated estimates of uncertainty (e.g. confidence intervals) |
| ☐ | ☒ | For null hypothesis testing, the test statistic (e.g. *F*, *t*, *r*) with confidence intervals, effect sizes, degrees of freedom and *P* value noted<br>*Give P values as exact values whenever suitable.* |
| ☒ | ☐ | For Bayesian analysis, information on the choice of priors and Markov chain Monte Carlo settings |
| ☒ | ☐ | For hierarchical and complex designs, identification of the appropriate level for tests and full reporting of outcomes |
| ☒ | ☐ | Estimates of effect sizes (e.g. Cohen's *d*, Pearson's *r*), indicating how they were calculated |

*Our web collection on statistics for biologists contains articles on many of the points above.*

## Software and code

Policy information about availability of computer code

| | |
|---|---|
| Data collection | cutadapt (version 2.4), BSMAP (version 2.90),GATK (version 4.1.4.1), MOABS (version 1.3.2), Nugene diversity adapter trimming (https://github.com/nugentechnologies/NuMetRRBS), NuDup (https://github.com/nugentechnologies/nudup), BWA (version 0.7.17), samtools (version 1.10), MACS2 (version 2.1.2), peakranger (version 1.18), deepTools (version 2.4.1), STAR (version 2.7.5a), stringtie (version 2.0.6), bowtie2 (version 2.3.5.1), HiCExplorer (version 3.6), Nanopype (version 1.1.0), guppy (v4.0.11), minimap2 (v2.10) , nanopolish (v0.13.2)<br><br>- Western blot images were collected using Image Lab software (version 6.1.0 build 7) (Bio-Rad)<br>- Single cell clones (ESC+TSC) and TSC knockout clones (GFP+) were sorted with FACS Diva software (BD Biosciences, v8.0.1) using BD FACS Aria II and BD FACSAria Fusion<br>- Brightfield images were collected using the Z1 Axio Observer and Zen Blue (2.3.69.1016) software (Zeiss) |
| Data analysis | R (version 3.6.3), metilene (version 0.2-8), bedtools (version 2.29.2), deepTools (version 2.4.1), pheatmap (version 1.0.12), EnrichedHeatmap (1.19.2), RLM (version 1.0.0), DESeq2 (version 1.26.0)<br>Custom code is available at 10.5281/zenodo.7492144.<br>- FACS data was analyzed with FlowJo (v10.7)<br>- GraphPad Prism (V 9.2.0) was used for the generation of bar plots and growth curves |

For manuscripts utilizing custom algorithms or software that are central to the research but not yet described in published literature, software must be made available to editors and reviewers. We strongly encourage code deposition in a community repository (e.g. GitHub). See the Nature Research guidelines for submitting code & software for further information.

## Data

Policy information about availability of data

All manuscripts must include a data availability statement. This statement should provide the following information, where applicable:

- Accession codes, unique identifiers, or web links for publicly available datasets
- A list of figures that have associated raw data
- A description of any restrictions on data availability

Sequencing data that support the findings of this study have been deposited in the Gene Expression Omnibus (GEO) under accession code GSE166362. Previously published data sets that were re-analyzed here are available under the following accession codes: WGBS data sets for wild type as well as Polycomb knockout mouse epiblast and extraembryonic ectoderm were obtained from GSE137337. WGBS for wild type mESCs was used from GSE158460. ChIP-seq for H3K27me3 profiled in mESCs and respective input samples were obtained from GSE116603, GSE120376 and GSE49847. mESC RNAseq replicates are available under GSE159468. WGBS of E15 and E18 mouse placental tissue were obtained from GSE84350. Source data are either provided with this study or available at 10.5281/zenodo.7492144. All other data supporting the findings of this study are available from the corresponding author on reasonable request.

# Field-specific reporting

Please select the one below that is the best fit for your research. If you are not sure, read the appropriate sections before making your selection.

☒ Life sciences ☐ Behavioural & social sciences ☐ Ecological, evolutionary & environmental sciences

For a reference copy of the document with all sections, see nature.com/documents/nr-reporting-summary-flat.pdf

# Life sciences study design

All studies must disclose on these points even when the disclosure is negative.

| | |
|---|---|
| Sample size | No statistical methods were used to predetermine sample sizes but our sample sizes are similar to those reported in previous publications (Smith et al. Nature 2017, Schoenfelder et al. Nature Communications 2018, Haggerty et al. Nature Structural and Molecular Biology 2021, Kumar et al. Nature Cell Biology 2022). Sample sizes are indicated in the figure panels or legends. |
| Data exclusions | No data was excluded. |
| Replication | Four different TSC lines from two different labs and including female and male lines were profiled in order to confirm that TSCs exhibit an intermediate, stochastic methylome similar to that of the extraembryonic ectoderm. Single Eed, Rnf2, Dnmt3b, Tet3 and Kdm2b knockouts were generated (no replicates) and the effect of the Eed knockout was verified using an inhibitor for EZH2. The effect of DNMT1i on TSCs was replicated in two different lines and three different experiments within the TSC1 line (at different passages). MINUTE-ChIP experiments were performed in triplicates. RNAseq, ChIP-BS-seq and EED ChIP-seq experiments were performed in duplicates. For RRBS, WGBS and Nanopore experiments single replicates per sample or time point were generated. Sex-typing and Western Blots were repeated at least three times, co-immunoprecipitation of EED and other proteins were repeated at least two times (one representative shown in this study). All attempts at replication were successful. |
| Randomization | Our genomic analyses are independent of human intervention and analyze each sample equally and in an unbiased fashion. For experiments, no pre-selection was done on experimental vs control samples during culture, treatment, library synthesis, or sequencing stages. |
| Blinding | Blinding was not relevant for this study since this is not an intervention study. However, our analytical pipeline followed uniform criteria applied to all samples, allowing us to analyze our data in an unbiased manner. |

# Reporting for specific materials, systems and methods

We require information from authors about some types of materials, experimental systems and methods used in many studies. Here, indicate whether each material, system or method listed is relevant to your study. If you are not sure if a list item applies to your research, read the appropriate section before selecting a response.

### Materials & experimental systems

| n/a | Involved in the study | |
|---|---|---|
| ☐ | ☒ | Antibodies |
| ☐ | ☒ | Eukaryotic cell lines |
| ☒ | ☐ | Palaeontology and archaeology |
| ☐ | ☒ | Animals and other organisms |
| ☒ | ☐ | Human research participants |
| ☒ | ☐ | Clinical data |
| ☒ | ☐ | Dual use research of concern |

### Methods

| n/a | Involved in the study | |
|---|---|---|
| ☐ | ☒ | ChIP-seq |
| ☐ | ☒ | Flow cytometry |
| ☒ | ☐ | MRI-based neuroimaging |

## Antibodies

Antibodies used

Supplier name, catalog number, clone/lot number
Western Blot and Co-IP/MS
Rabbit anti-H3K27me3: Cell Signaling Technology, #9733S, C36B11
Rabbit anti-H2AK119Ub1: Cell Signaling Technology, #8240S, D27C4
Rabbit anti-Histone H4: Cell Signaling Technology, #2592
Mouse anti-Rabbit-IgG HRP: Jackson, cat# 211-032-171, clone: 5A6-1D10
Rabbit anti-IgG: Cell Signaling Technology, #2729, lot:10
Rabbit anti-EED: Abcam, cat# ab4469, lot:GR3207387-1
Rabbit anti-Rnf2: Cell Signaling Technology, #5694, clone: D22F2
Rabbit anti-DNMT3B: Cell Signaling Technology, #48488S, clone: E4I40
Rabbit anti-RYBP: Millipore, #AB3637
Rabbit anti-Suz12: Cell Signaling Technology, #3737T, clone: 8
Mouse anti-Tubulin: Santa Cruz, #sc-32293, clone: DM1A
MINUTE ChIP:
Rabbit anti-H3K4me3: Millipore, #04-745, clone MC315
Rabbit anti-H3K27me3: Cell Signaling Technology, #9733, clone: C36B11
Rabbit anti-H2AK119ub: Cell Signaling Technology, #8240S, clone: D27C4
ChIP-BS-seq:
Rabbit anti-H3K27me3: Thermo Fisher, MA5-11198, lot: WH3366172
ChIP-seq:
Rabbit anti-EED: Abcam, ab240650, EPR23043-5, lot: GR3427609-2

Validation

Antibodies were validated by comparing immunofluorescence data and western blot in wild type and knockout trophoblast stem cells (data not shown). Additionally, all antibodies were validated by the provider and cited in numerous publications (information below)

The rabbit anti-H3K27me3 (CST) antibody has been validated using SimpleChIP® Enzymatic Chromatin IP Kits.
https://www.cellsignal.de/products/primary-antibodies/tri-methyl-histone-h3-lys27-c36b11-rabbit-mab/9733

The rabbit anti-H2AK119Ub1 antibody has been validated using SimpleChIP® Enzymatic Chromatin IP Kits.
https://www.cellsignal.com/products/primary-antibodies/ubiquityl-histone-h2a-lys119-d27c4-xp-rabbit-mab/8240

The rabbit anti-Histone H4 antibody has been validated by WB.
https://www.cellsignal.de/products/primary-antibodies/histone-h4-antibody/2592

The rabbit anti-IgG antibody has been validated by ChIP and IP.
https://www.cellsignal.de/products/primary-antibodies/normal-rabbit-igg/2729

The rabbit anti-EED (abcam, #ab4469) antibody has been validated for WB and ICC/IF. This antibody is additionally knockout validated.
https://www.abcam.com/eed-antibody-ab4469.html

The rabbit anti-Rnf2 antibody has been validated using SimpleChIP® Enzymatic Chromatin IP Kits.
https://www.cellsignal.de/products/primary-antibodies/ring1b-d22f2-xp-rabbit-mab/5694

The rabbit anti-DNMT3B antibody is highly specific and rigorously validated by the provider for ChIP, IP, WB and IF.
https://www.cellsignal.com/products/primary-antibodies/dnmt3b-e4i4o-rabbit-mab-mouse-specific/48488

The rabbit anti-RYBP antibody has been validated for use in WB.
https://www.merckmillipore.com/DE/de/product/Anti-DEDAF-Antibody,MM_NF-AB3637?ReferrerURL=https%3A%2F%2Fwww.google.com%2F

The rabbit anti-Suz12 antibody has been validated using SimpleChIP® Enzymatic Chromatin IP Kits.
https://www.cellsignal.com/products/primary-antibodies/suz12-d39f6-xp-rabbit-mab/3737

The mouse anti-Tubulin antibody has been validated for WB and IF.
https://www.scbt.com/p/alpha-tubulin-antibody-dm1a?productCanUrl=alpha-tubulin-antibody-dm1a&_requestid=1049815

The rabbit anti-H3K4me3 antibody has been validated for WB, ChIP, DB, Mplex and ChIP-seq.
https://www.merckmillipore.com/DE/de/product/Anti-trimethyl-Histone-H3-Lys4-Antibody-clone-MC315-rabbit-monoclonal,MM_NF-04-745

The rabbit anti-H3K27me3 (Thermo Fisher) antibody has been extensively validated for specificity using SNAP-ChIP™ spike-in and peptide array.
https://www.thermofisher.com/antibody/product/H3K27me3-Antibody-clone-G-299-10-Monoclonal/MA5-11198

The rabbit anti-EED (abcam, #ab240650) antibody has been validated in WB, ChIP-seq and IP. This antibody is additionally knockout validated.
https://www.abcam.com/eed-antibody-epr23043-5-chip-grade-ab240650.html

# Eukaryotic cell lines

Policy information about cell lines

| | |
|---|---|
| Cell line source(s) | Trophoblast Stem Cell (TSC) lines were derived from CD-1 strain blastocysts. Pronuclei Stage 3 (PN3) zygotes were isolated from natural mating of CD-1 strain mice (Charles river). More detailed info is provided in Methods under: "Derivation of Trophoblast stem cells".<br>V6.5 mouse embryonic stem cell line (mESCs), source: Konrad Hochedlinger lab |
| Authentication | The identity of V6.5 mESCs and mTSCs including all cell lines derived from them have been validated using morphological characteristics, immunofluorescence, marker gene expression (RNAseq), but have not been authenticated. Knockout cell lines were validated by genotyping, western blotting, Sanger sequencing and next generation sequencing (RNAseq/RRBS/WGBS). |
| Mycoplasma contamination | All cell lines are negative for mycoplasma contamination and were regularly tested throughout the study. |
| Commonly misidentified lines<br>(See ICLAC register) | No commonly misidentified cell lines were used. |

# Animals and other organisms

Policy information about studies involving animals; ARRIVE guidelines recommended for reporting animal research

| | |
|---|---|
| Laboratory animals | CD-1 strain Mus musculus domesticus animals (male and female) were used to generate blastocysts for TSC derivation. |
| Wild animals | *Provide details on animals observed in or captured in the field; report species, sex and age where possible. Describe how animals were caught and transported and what happened to captive animals after the study (if killed, explain why and describe method; if released, say where and when) OR state that the study did not involve wild animals.* |
| Field-collected samples | *For laboratory work with field-collected samples, describe all relevant parameters such as housing, maintenance, temperature, photoperiod and end-of-experiment protocol OR state that the study did not involve samples collected from the field.* |
| Ethics oversight | All research described here complies with the relevant ethical regulations at the respective institutions. Work at the Max Planck Institute was approved by the LAGESO. |

Note that full information on the approval of the study protocol must also be provided in the manuscript.

# ChIP-seq

## Data deposition

☒ Confirm that both raw and final processed data have been deposited in a public database such as GEO.

☒ Confirm that you have deposited or provided access to graph files (e.g. BED files) for the called peaks.

| | |
|---|---|
| Data access links<br>*May remain private before publication.* | Datasets generated in this study have been deposited in the Gene Expression Omnibus under accession number GSE166362. |
| Files in database submission | MINUTE_ESC_WT_H2AK119ub1_Pool1_Rep1_R1.fastq.gz MINUTE_ESC_WT_H2AK119ub1_Pool1_Rep1_R2.fastq.gz<br>MINUTE_ESC_WT_H2AK119ub1_Pool1_Rep2_R1.fastq.gz MINUTE_ESC_WT_H2AK119ub1_Pool1_Rep2_R2.fastq.gz<br>MINUTE_ESC_WT_H2AK119ub1_Pool1_Rep3_R1.fastq.gz MINUTE_ESC_WT_H2AK119ub1_Pool1_Rep3_R2.fastq.gz<br>MINUTE_ESC_WT_H3K27me3_Pool1_Rep1_R1.fastq.gz MINUTE_ESC_WT_H3K27me3_Pool1_Rep1_R2.fastq.gz<br>MINUTE_ESC_WT_H3K27me3_Pool1_Rep2_R1.fastq.gz MINUTE_ESC_WT_H3K27me3_Pool1_Rep2_R2.fastq.gz<br>MINUTE_ESC_WT_H3K27me3_Pool1_Rep3_R1.fastq.gz MINUTE_ESC_WT_H3K27me3_Pool1_Rep3_R2.fastq.gz<br>MINUTE_ESC_WT_H3K4me3_Pool1_Rep1_R1.fastq.gz MINUTE_ESC_WT_H3K4me3_Pool1_Rep1_R2.fastq.gz<br>MINUTE_ESC_WT_H3K4me3_Pool1_Rep2_R1.fastq.gz MINUTE_ESC_WT_H3K4me3_Pool1_Rep2_R2.fastq.gz<br>MINUTE_ESC_WT_H3K4me3_Pool1_Rep3_R1.fastq.gz MINUTE_ESC_WT_H3K4me3_Pool1_Rep3_R2.fastq.gz<br>MINUTE_ESC_WT_Input_Pool1_Rep1_R1.fastq.gz MINUTE_ESC_WT_Input_Pool1_Rep1_R2.fastq.gz<br>MINUTE_ESC_WT_Input_Pool1_Rep2_R1.fastq.gz MINUTE_ESC_WT_Input_Pool1_Rep2_R2.fastq.gz<br>MINUTE_ESC_WT_Input_Pool1_Rep3_R1.fastq.gz MINUTE_ESC_WT_Input_Pool1_Rep3_R2.fastq.gz<br>MINUTE_TSC1_KDM2BKO_H2AK119ub1_Pool2_Rep1_R1.fastq.gz<br>MINUTE_TSC1_KDM2BKO_H2AK119ub1_Pool2_Rep1_R2.fastq.gz<br>MINUTE_TSC1_KDM2BKO_H2AK119ub1_Pool2_Rep2_R1.fastq.gz<br>MINUTE_TSC1_KDM2BKO_H2AK119ub1_Pool2_Rep2_R2.fastq.gz<br>MINUTE_TSC1_KDM2BKO_H2AK119ub1_Pool2_Rep3_R1.fastq.gz<br>MINUTE_TSC1_KDM2BKO_H2AK119ub1_Pool2_Rep3_R2.fastq.gz<br>MINUTE_TSC1_KDM2BKO_H3K27me3_Pool2_Rep1_R1.fastq.gz<br>MINUTE_TSC1_KDM2BKO_H3K27me3_Pool2_Rep1_R2.fastq.gz<br>MINUTE_TSC1_KDM2BKO_H3K27me3_Pool2_Rep2_R1.fastq.gz<br>MINUTE_TSC1_KDM2BKO_H3K27me3_Pool2_Rep2_R2.fastq.gz<br>MINUTE_TSC1_KDM2BKO_H3K27me3_Pool2_Rep3_R1.fastq.gz<br>MINUTE_TSC1_KDM2BKO_H3K27me3_Pool2_Rep3_R2.fastq.gz<br>MINUTE_TSC1_KDM2BKO_H3K4me3_Pool2_Rep1_R1.fastq.gz MINUTE_TSC1_KDM2BKO_H3K4me3_Pool2_Rep1_R2.fastq.gz |

MINUTE_TSC1_KDM2BKO_H3K4me3_Pool2_Rep2_R1.fastq.gz MINUTE_TSC1_KDM2BKO_H3K4me3_Pool2_Rep2_R2.fastq.gz
MINUTE_TSC1_KDM2BKO_H3K4me3_Pool2_Rep3_R1.fastq.gz MINUTE_TSC1_KDM2BKO_H3K4me3_Pool2_Rep3_R2.fastq.gz
MINUTE_TSC1_KDM2BKO_Input_Pool2_Rep1_R1.fastq.gz MINUTE_TSC1_KDM2BKO_Input_Pool2_Rep1_R2.fastq.gz
MINUTE_TSC1_KDM2BKO_Input_Pool2_Rep2_R1.fastq.gz MINUTE_TSC1_KDM2BKO_Input_Pool2_Rep2_R2.fastq.gz
MINUTE_TSC1_KDM2BKO_Input_Pool2_Rep3_R1.fastq.gz MINUTE_TSC1_KDM2BKO_Input_Pool2_Rep3_R2.fastq.gz
MINUTE_TSC1_RNF2KO_H2AK119ub1_Pool2_Rep1_R1.fastq.gz
MINUTE_TSC1_RNF2KO_H2AK119ub1_Pool2_Rep1_R2.fastq.gz
MINUTE_TSC1_RNF2KO_H2AK119ub1_Pool2_Rep2_R1.fastq.gz
MINUTE_TSC1_RNF2KO_H2AK119ub1_Pool2_Rep2_R2.fastq.gz
MINUTE_TSC1_RNF2KO_H2AK119ub1_Pool2_Rep3_R1.fastq.gz
MINUTE_TSC1_RNF2KO_H2AK119ub1_Pool2_Rep3_R2.fastq.gz
MINUTE_TSC1_RNF2KO_H3K27me3_Pool2_Rep1_R1.fastq.gz MINUTE_TSC1_RNF2KO_H3K27me3_Pool2_Rep1_R2.fastq.gz
MINUTE_TSC1_RNF2KO_H3K27me3_Pool2_Rep2_R1.fastq.gz MINUTE_TSC1_RNF2KO_H3K27me3_Pool2_Rep2_R2.fastq.gz
MINUTE_TSC1_RNF2KO_H3K27me3_Pool2_Rep3_R1.fastq.gz MINUTE_TSC1_RNF2KO_H3K27me3_Pool2_Rep3_R2.fastq.gz
MINUTE_TSC1_RNF2KO_H3K4me3_Pool2_Rep1_R1.fastq.gz MINUTE_TSC1_RNF2KO_H3K4me3_Pool2_Rep1_R2.fastq.gz
MINUTE_TSC1_RNF2KO_H3K4me3_Pool2_Rep2_R1.fastq.gz MINUTE_TSC1_RNF2KO_H3K4me3_Pool2_Rep2_R2.fastq.gz
MINUTE_TSC1_RNF2KO_H3K4me3_Pool2_Rep3_R1.fastq.gz MINUTE_TSC1_RNF2KO_H3K4me3_Pool2_Rep3_R2.fastq.gz
MINUTE_TSC1_RNF2KO_Input_Pool2_Rep1_R1.fastq.gz MINUTE_TSC1_RNF2KO_Input_Pool2_Rep1_R2.fastq.gz
MINUTE_TSC1_RNF2KO_Input_Pool2_Rep2_R1.fastq.gz MINUTE_TSC1_RNF2KO_Input_Pool2_Rep2_R2.fastq.gz
MINUTE_TSC1_RNF2KO_Input_Pool2_Rep3_R1.fastq.gz MINUTE_TSC1_RNF2KO_Input_Pool2_Rep3_R2.fastq.gz
MINUTE_TSC1_WT_EZH2i_5w_recovery_4w_H2AK119ub1_Pool2_Rep1_R1.fastq.gz
MINUTE_TSC1_WT_EZH2i_5w_recovery_4w_H2AK119ub1_Pool2_Rep1_R2.fastq.gz
MINUTE_TSC1_WT_EZH2i_5w_recovery_4w_H2AK119ub1_Pool2_Rep2_R1.fastq.gz
MINUTE_TSC1_WT_EZH2i_5w_recovery_4w_H2AK119ub1_Pool2_Rep2_R2.fastq.gz
MINUTE_TSC1_WT_EZH2i_5w_recovery_4w_H2AK119ub1_Pool2_Rep3_R1.fastq.gz
MINUTE_TSC1_WT_EZH2i_5w_recovery_4w_H2AK119ub1_Pool2_Rep3_R2.fastq.gz
MINUTE_TSC1_WT_EZH2i_5w_recovery_4w_H3K27me3_Pool2_Rep1_R1.fastq.gz
MINUTE_TSC1_WT_EZH2i_5w_recovery_4w_H3K27me3_Pool2_Rep1_R2.fastq.gz
MINUTE_TSC1_WT_EZH2i_5w_recovery_4w_H3K27me3_Pool2_Rep2_R1.fastq.gz
MINUTE_TSC1_WT_EZH2i_5w_recovery_4w_H3K27me3_Pool2_Rep2_R2.fastq.gz
MINUTE_TSC1_WT_EZH2i_5w_recovery_4w_H3K27me3_Pool2_Rep3_R1.fastq.gz
MINUTE_TSC1_WT_EZH2i_5w_recovery_4w_H3K27me3_Pool2_Rep3_R2.fastq.gz
MINUTE_TSC1_WT_EZH2i_5w_recovery_4w_H3K4me3_Pool2_Rep1_R1.fastq.gz
MINUTE_TSC1_WT_EZH2i_5w_recovery_4w_H3K4me3_Pool2_Rep1_R2.fastq.gz
MINUTE_TSC1_WT_EZH2i_5w_recovery_4w_H3K4me3_Pool2_Rep2_R1.fastq.gz
MINUTE_TSC1_WT_EZH2i_5w_recovery_4w_H3K4me3_Pool2_Rep2_R2.fastq.gz
MINUTE_TSC1_WT_EZH2i_5w_recovery_4w_H3K4me3_Pool2_Rep3_R1.fastq.gz
MINUTE_TSC1_WT_EZH2i_5w_recovery_4w_H3K4me3_Pool2_Rep3_R2.fastq.gz
MINUTE_TSC1_WT_EZH2i_5w_recovery_4w_Input_Pool2_Rep1_R1.fastq.gz
MINUTE_TSC1_WT_EZH2i_5w_recovery_4w_Input_Pool2_Rep1_R2.fastq.gz
MINUTE_TSC1_WT_EZH2i_5w_recovery_4w_Input_Pool2_Rep2_R1.fastq.gz
MINUTE_TSC1_WT_EZH2i_5w_recovery_4w_Input_Pool2_Rep2_R2.fastq.gz
MINUTE_TSC1_WT_EZH2i_5w_recovery_4w_Input_Pool2_Rep3_R1.fastq.gz
MINUTE_TSC1_WT_EZH2i_5w_recovery_4w_Input_Pool2_Rep3_R2.fastq.gz
MINUTE_TSC1_WT_H2AK119ub1_Pool1_Rep1_R1.fastq.gz MINUTE_TSC1_WT_H2AK119ub1_Pool1_Rep1_R2.fastq.gz
MINUTE_TSC1_WT_H2AK119ub1_Pool1_Rep2_R1.fastq.gz MINUTE_TSC1_WT_H2AK119ub1_Pool1_Rep2_R2.fastq.gz
MINUTE_TSC1_WT_H2AK119ub1_Pool1_Rep3_R1.fastq.gz MINUTE_TSC1_WT_H2AK119ub1_Pool1_Rep3_R2.fastq.gz
MINUTE_TSC1_WT_H2AK119ub1_Pool2_Rep1_R1.fastq.gz MINUTE_TSC1_WT_H2AK119ub1_Pool2_Rep1_R2.fastq.gz
MINUTE_TSC1_WT_H2AK119ub1_Pool2_Rep2_R1.fastq.gz MINUTE_TSC1_WT_H2AK119ub1_Pool2_Rep2_R2.fastq.gz
MINUTE_TSC1_WT_H2AK119ub1_Pool2_Rep3_R1.fastq.gz MINUTE_TSC1_WT_H2AK119ub1_Pool2_Rep3_R2.fastq.gz
MINUTE_TSC1_WT_H3K27me3_Pool1_Rep1_R1.fastq.gz MINUTE_TSC1_WT_H3K27me3_Pool1_Rep1_R2.fastq.gz
MINUTE_TSC1_WT_H3K27me3_Pool1_Rep2_R1.fastq.gz MINUTE_TSC1_WT_H3K27me3_Pool1_Rep2_R2.fastq.gz
MINUTE_TSC1_WT_H3K27me3_Pool1_Rep3_R1.fastq.gz MINUTE_TSC1_WT_H3K27me3_Pool1_Rep3_R2.fastq.gz
MINUTE_TSC1_WT_H3K27me3_Pool2_Rep1_R1.fastq.gz MINUTE_TSC1_WT_H3K27me3_Pool2_Rep1_R2.fastq.gz
MINUTE_TSC1_WT_H3K27me3_Pool2_Rep2_R1.fastq.gz MINUTE_TSC1_WT_H3K27me3_Pool2_Rep2_R2.fastq.gz
MINUTE_TSC1_WT_H3K27me3_Pool2_Rep3_R1.fastq.gz MINUTE_TSC1_WT_H3K27me3_Pool2_Rep3_R2.fastq.gz
MINUTE_TSC1_WT_H3K4me3_Pool1_Rep1_R1.fastq.gz MINUTE_TSC1_WT_H3K4me3_Pool1_Rep1_R2.fastq.gz
MINUTE_TSC1_WT_H3K4me3_Pool1_Rep2_R1.fastq.gz MINUTE_TSC1_WT_H3K4me3_Pool1_Rep2_R2.fastq.gz
MINUTE_TSC1_WT_H3K4me3_Pool1_Rep3_R1.fastq.gz MINUTE_TSC1_WT_H3K4me3_Pool1_Rep3_R2.fastq.gz
MINUTE_TSC1_WT_H3K4me3_Pool2_Rep1_R1.fastq.gz MINUTE_TSC1_WT_H3K4me3_Pool2_Rep1_R2.fastq.gz
MINUTE_TSC1_WT_H3K4me3_Pool2_Rep2_R1.fastq.gz MINUTE_TSC1_WT_H3K4me3_Pool2_Rep2_R2.fastq.gz
MINUTE_TSC1_WT_H3K4me3_Pool2_Rep3_R1.fastq.gz MINUTE_TSC1_WT_H3K4me3_Pool2_Rep3_R2.fastq.gz
MINUTE_TSC1_WT_Input_Pool1_Rep1_R1.fastq.gz MINUTE_TSC1_WT_Input_Pool1_Rep1_R2.fastq.gz
MINUTE_TSC1_WT_Input_Pool1_Rep2_R1.fastq.gz MINUTE_TSC1_WT_Input_Pool1_Rep2_R2.fastq.gz
MINUTE_TSC1_WT_Input_Pool1_Rep3_R1.fastq.gz MINUTE_TSC1_WT_Input_Pool1_Rep3_R2.fastq.gz
MINUTE_TSC1_WT_Input_Pool2_Rep1_R1.fastq.gz MINUTE_TSC1_WT_Input_Pool2_Rep1_R2.fastq.gz
MINUTE_TSC1_WT_Input_Pool2_Rep2_R1.fastq.gz MINUTE_TSC1_WT_Input_Pool2_Rep2_R2.fastq.gz
MINUTE_TSC1_WT_Input_Pool2_Rep3_R1.fastq.gz MINUTE_TSC1_WT_Input_Pool2_Rep3_R2.fastq.gz
MINUTE_TSC2_3BKO_H2AK119ub1_Pool1_Rep1_R1.fastq.gz MINUTE_TSC2_3BKO_H2AK119ub1_Pool1_Rep1_R2.fastq.gz
MINUTE_TSC2_3BKO_H2AK119ub1_Pool1_Rep2_R1.fastq.gz MINUTE_TSC2_3BKO_H2AK119ub1_Pool1_Rep2_R2.fastq.gz
MINUTE_TSC2_3BKO_H2AK119ub1_Pool1_Rep3_R1.fastq.gz MINUTE_TSC2_3BKO_H2AK119ub1_Pool1_Rep3_R2.fastq.gz
MINUTE_TSC2_3BKO_H3K27me3_Pool1_Rep1_R1.fastq.gz MINUTE_TSC2_3BKO_H3K27me3_Pool1_Rep1_R2.fastq.gz
MINUTE_TSC2_3BKO_H3K27me3_Pool1_Rep2_R1.fastq.gz MINUTE_TSC2_3BKO_H3K27me3_Pool1_Rep2_R2.fastq.gz
MINUTE_TSC2_3BKO_H3K27me3_Pool1_Rep3_R1.fastq.gz MINUTE_TSC2_3BKO_H3K27me3_Pool1_Rep3_R2.fastq.gz
MINUTE_TSC2_3BKO_H3K4me3_Pool1_Rep1_R1.fastq.gz MINUTE_TSC2_3BKO_H3K4me3_Pool1_Rep1_R2.fastq.gz
MINUTE_TSC2_3BKO_H3K4me3_Pool1_Rep2_R1.fastq.gz MINUTE_TSC2_3BKO_H3K4me3_Pool1_Rep2_R2.fastq.gz

MINUTE_TSC2_3BKO_H3K4me3_Pool1_Rep3_R1.fastq.gz MINUTE_TSC2_3BKO_H3K4me3_Pool1_Rep3_R2.fastq.gz
MINUTE_TSC2_3BKO_Input_Pool1_Rep1_R1.fastq.gz MINUTE_TSC2_3BKO_Input_Pool1_Rep1_R2.fastq.gz
MINUTE_TSC2_3BKO_Input_Pool1_Rep2_R1.fastq.gz MINUTE_TSC2_3BKO_Input_Pool1_Rep2_R2.fastq.gz
MINUTE_TSC2_3BKO_Input_Pool1_Rep3_R1.fastq.gz MINUTE_TSC2_3BKO_Input_Pool1_Rep3_R2.fastq.gz
MINUTE_TSC3_EEDKO_H2AK119ub1_Pool2_Rep1_R1.fastq.gz MINUTE_TSC3_EEDKO_H2AK119ub1_Pool2_Rep1_R2.fastq.gz
MINUTE_TSC3_EEDKO_H2AK119ub1_Pool2_Rep2_R1.fastq.gz MINUTE_TSC3_EEDKO_H2AK119ub1_Pool2_Rep2_R2.fastq.gz
MINUTE_TSC3_EEDKO_H2AK119ub1_Pool2_Rep3_R1.fastq.gz MINUTE_TSC3_EEDKO_H2AK119ub1_Pool2_Rep3_R2.fastq.gz
MINUTE_TSC3_EEDKO_H3K27me3_Pool2_Rep1_R1.fastq.gz MINUTE_TSC3_EEDKO_H3K27me3_Pool2_Rep1_R2.fastq.gz
MINUTE_TSC3_EEDKO_H3K27me3_Pool2_Rep2_R1.fastq.gz MINUTE_TSC3_EEDKO_H3K27me3_Pool2_Rep2_R2.fastq.gz
MINUTE_TSC3_EEDKO_H3K27me3_Pool2_Rep3_R1.fastq.gz MINUTE_TSC3_EEDKO_H3K27me3_Pool2_Rep3_R2.fastq.gz
MINUTE_TSC3_EEDKO_H3K4me3_Pool2_Rep1_R1.fastq.gz MINUTE_TSC3_EEDKO_H3K4me3_Pool2_Rep1_R2.fastq.gz
MINUTE_TSC3_EEDKO_H3K4me3_Pool2_Rep2_R1.fastq.gz MINUTE_TSC3_EEDKO_H3K4me3_Pool2_Rep2_R2.fastq.gz
MINUTE_TSC3_EEDKO_H3K4me3_Pool2_Rep3_R1.fastq.gz MINUTE_TSC3_EEDKO_H3K4me3_Pool2_Rep3_R2.fastq.gz
MINUTE_TSC3_EEDKO_Input_Pool2_Rep1_R1.fastq.gz MINUTE_TSC3_EEDKO_Input_Pool2_Rep1_R2.fastq.gz
MINUTE_TSC3_EEDKO_Input_Pool2_Rep2_R1.fastq.gz MINUTE_TSC3_EEDKO_Input_Pool2_Rep2_R2.fastq.gz
MINUTE_TSC3_EEDKO_Input_Pool2_Rep3_R1.fastq.gz MINUTE_TSC3_EEDKO_Input_Pool2_Rep3_R2.fastq.gz
MINUTE_ESC_WT_H2AK119ub1_Pool1_Rep1_RPGC_mm10_scaled.bw
MINUTE_ESC_WT_H2AK119ub1_Pool1_Rep2_RPGC_mm10_scaled.bw
MINUTE_ESC_WT_H2AK119ub1_Pool1_Rep3_RPGC_mm10_scaled.bw
MINUTE_ESC_WT_H3K27me3_Pool1_Rep1_RPGC_mm10_scaled.bw
MINUTE_ESC_WT_H3K27me3_Pool1_Rep2_RPGC_mm10_scaled.bw
MINUTE_ESC_WT_H3K27me3_Pool1_Rep3_RPGC_mm10_scaled.bw
MINUTE_ESC_WT_H3K4me3_Pool1_Rep1_RPGC_mm10_scaled.bw
MINUTE_ESC_WT_H3K4me3_Pool1_Rep2_RPGC_mm10_scaled.bw
MINUTE_ESC_WT_H3K4me3_Pool1_Rep3_RPGC_mm10_scaled.bw
MINUTE_ESC_WT_Input_Pool1_Rep1_RPGC_mm10_unscaled.bw
MINUTE_ESC_WT_Input_Pool1_Rep2_RPGC_mm10_unscaled.bw
MINUTE_ESC_WT_Input_Pool1_Rep3_RPGC_mm10_unscaled.bw
MINUTE_TSC1_KDM2BKO_H2AK119ub1_Pool2_Rep1_RPGC_mm10_scaled.bw
MINUTE_TSC1_KDM2BKO_H2AK119ub1_Pool2_Rep2_RPGC_mm10_scaled.bw
MINUTE_TSC1_KDM2BKO_H2AK119ub1_Pool2_Rep3_RPGC_mm10_scaled.bw
MINUTE_TSC1_KDM2BKO_H3K27me3_Pool2_Rep1_RPGC_mm10_scaled.bw
MINUTE_TSC1_KDM2BKO_H3K27me3_Pool2_Rep2_RPGC_mm10_scaled.bw
MINUTE_TSC1_KDM2BKO_H3K27me3_Pool2_Rep3_RPGC_mm10_scaled.bw
MINUTE_TSC1_KDM2BKO_H3K4me3_Pool2_Rep1_RPGC_mm10_scaled.bw
MINUTE_TSC1_KDM2BKO_H3K4me3_Pool2_Rep2_RPGC_mm10_scaled.bw
MINUTE_TSC1_KDM2BKO_H3K4me3_Pool2_Rep3_RPGC_mm10_scaled.bw
MINUTE_TSC1_KDM2BKO_Input_Pool2_Rep1_RPGC_mm10_unscaled.bw
MINUTE_TSC1_KDM2BKO_Input_Pool2_Rep2_RPGC_mm10_unscaled.bw
MINUTE_TSC1_KDM2BKO_Input_Pool2_Rep3_RPGC_mm10_unscaled.bw
MINUTE_TSC1_RNF2KO_H2AK119ub1_Pool2_Rep1_RPGC_mm10_scaled.bw
MINUTE_TSC1_RNF2KO_H2AK119ub1_Pool2_Rep2_RPGC_mm10_scaled.bw
MINUTE_TSC1_RNF2KO_H2AK119ub1_Pool2_Rep3_RPGC_mm10_scaled.bw
MINUTE_TSC1_RNF2KO_H3K27me3_Pool2_Rep1_RPGC_mm10_scaled.bw
MINUTE_TSC1_RNF2KO_H3K27me3_Pool2_Rep2_RPGC_mm10_scaled.bw
MINUTE_TSC1_RNF2KO_H3K27me3_Pool2_Rep3_RPGC_mm10_scaled.bw
MINUTE_TSC1_RNF2KO_H3K4me3_Pool2_Rep1_RPGC_mm10_scaled.bw
MINUTE_TSC1_RNF2KO_H3K4me3_Pool2_Rep2_RPGC_mm10_scaled.bw
MINUTE_TSC1_RNF2KO_H3K4me3_Pool2_Rep3_RPGC_mm10_scaled.bw
MINUTE_TSC1_RNF2KO_Input_Pool2_Rep1_RPGC_mm10_unscaled.bw
MINUTE_TSC1_RNF2KO_Input_Pool2_Rep2_RPGC_mm10_unscaled.bw
MINUTE_TSC1_RNF2KO_Input_Pool2_Rep3_RPGC_mm10_unscaled.bw
MINUTE_TSC1_WT_EZH2i_5w_recovery_4w_H2AK119ub1_Pool2_Rep1_RPGC_mm10_scaled.bw
MINUTE_TSC1_WT_EZH2i_5w_recovery_4w_H2AK119ub1_Pool2_Rep2_RPGC_mm10_scaled.bw
MINUTE_TSC1_WT_EZH2i_5w_recovery_4w_H2AK119ub1_Pool2_Rep3_RPGC_mm10_scaled.bw
MINUTE_TSC1_WT_EZH2i_5w_recovery_4w_H3K27me3_Pool2_Rep1_RPGC_mm10_scaled.bw
MINUTE_TSC1_WT_EZH2i_5w_recovery_4w_H3K27me3_Pool2_Rep2_RPGC_mm10_scaled.bw
MINUTE_TSC1_WT_EZH2i_5w_recovery_4w_H3K27me3_Pool2_Rep3_RPGC_mm10_scaled.bw
MINUTE_TSC1_WT_EZH2i_5w_recovery_4w_H3K4me3_Pool2_Rep1_RPGC_mm10_scaled.bw
MINUTE_TSC1_WT_EZH2i_5w_recovery_4w_H3K4me3_Pool2_Rep2_RPGC_mm10_scaled.bw
MINUTE_TSC1_WT_EZH2i_5w_recovery_4w_H3K4me3_Pool2_Rep3_RPGC_mm10_scaled.bw
MINUTE_TSC1_WT_EZH2i_5w_recovery_4w_Input_Pool2_Rep1_RPGC_mm10_unscaled.bw
MINUTE_TSC1_WT_EZH2i_5w_recovery_4w_Input_Pool2_Rep2_RPGC_mm10_unscaled.bw
MINUTE_TSC1_WT_EZH2i_5w_recovery_4w_Input_Pool2_Rep3_RPGC_mm10_unscaled.bw
MINUTE_TSC1_WT_H2AK119ub1_Pool1_Rep1_RPGC_mm10_scaled.bw
MINUTE_TSC1_WT_H2AK119ub1_Pool1_Rep2_RPGC_mm10_scaled.bw
MINUTE_TSC1_WT_H2AK119ub1_Pool1_Rep3_RPGC_mm10_scaled.bw
MINUTE_TSC1_WT_H2AK119ub1_Pool2_Rep1_RPGC_mm10_scaled.bw
MINUTE_TSC1_WT_H2AK119ub1_Pool2_Rep2_RPGC_mm10_scaled.bw
MINUTE_TSC1_WT_H2AK119ub1_Pool2_Rep3_RPGC_mm10_scaled.bw
MINUTE_TSC1_WT_H3K27me3_Pool1_Rep1_RPGC_mm10_scaled.bw
MINUTE_TSC1_WT_H3K27me3_Pool1_Rep2_RPGC_mm10_scaled.bw
MINUTE_TSC1_WT_H3K27me3_Pool1_Rep3_RPGC_mm10_scaled.bw
MINUTE_TSC1_WT_H3K27me3_Pool2_Rep1_RPGC_mm10_scaled.bw
MINUTE_TSC1_WT_H3K27me3_Pool2_Rep2_RPGC_mm10_scaled.bw
MINUTE_TSC1_WT_H3K27me3_Pool2_Rep3_RPGC_mm10_scaled.bw

MINUTE_TSC1_WT_H3K4me3_Pool1_Rep1_RPGC_mm10_scaled.bw
MINUTE_TSC1_WT_H3K4me3_Pool1_Rep2_RPGC_mm10_scaled.bw
MINUTE_TSC1_WT_H3K4me3_Pool1_Rep3_RPGC_mm10_scaled.bw
MINUTE_TSC1_WT_H3K4me3_Pool2_Rep1_RPGC_mm10_scaled.bw
MINUTE_TSC1_WT_H3K4me3_Pool2_Rep2_RPGC_mm10_scaled.bw
MINUTE_TSC1_WT_H3K4me3_Pool2_Rep3_RPGC_mm10_scaled.bw
MINUTE_TSC1_WT_Input_Pool1_Rep1_RPGC_mm10_unscaled.bw
MINUTE_TSC1_WT_Input_Pool1_Rep2_RPGC_mm10_unscaled.bw
MINUTE_TSC1_WT_Input_Pool1_Rep3_RPGC_mm10_unscaled.bw
MINUTE_TSC1_WT_Input_Pool2_Rep1_RPGC_mm10_unscaled.bw
MINUTE_TSC1_WT_Input_Pool2_Rep2_RPGC_mm10_unscaled.bw
MINUTE_TSC1_WT_Input_Pool2_Rep3_RPGC_mm10_unscaled.bw
MINUTE_TSC2_3BKO_H2AK119ub1_Pool1_Rep1_RPGC_mm10_scaled.bw
MINUTE_TSC2_3BKO_H2AK119ub1_Pool1_Rep2_RPGC_mm10_scaled.bw
MINUTE_TSC2_3BKO_H2AK119ub1_Pool1_Rep3_RPGC_mm10_scaled.bw
MINUTE_TSC2_3BKO_H3K27me3_Pool1_Rep1_RPGC_mm10_scaled.bw
MINUTE_TSC2_3BKO_H3K27me3_Pool1_Rep2_RPGC_mm10_scaled.bw
MINUTE_TSC2_3BKO_H3K27me3_Pool1_Rep3_RPGC_mm10_scaled.bw
MINUTE_TSC2_3BKO_H3K4me3_Pool1_Rep1_RPGC_mm10_scaled.bw
MINUTE_TSC2_3BKO_H3K4me3_Pool1_Rep2_RPGC_mm10_scaled.bw
MINUTE_TSC2_3BKO_H3K4me3_Pool1_Rep3_RPGC_mm10_scaled.bw
MINUTE_TSC2_3BKO_Input_Pool1_Rep1_RPGC_mm10_unscaled.bw
MINUTE_TSC2_3BKO_Input_Pool1_Rep2_RPGC_mm10_unscaled.bw
MINUTE_TSC2_3BKO_Input_Pool1_Rep3_RPGC_mm10_unscaled.bw
MINUTE_TSC3_EEDKO_H2AK119ub1_Pool2_Rep1_RPGC_mm10_scaled.bw
MINUTE_TSC3_EEDKO_H2AK119ub1_Pool2_Rep2_RPGC_mm10_scaled.bw
MINUTE_TSC3_EEDKO_H2AK119ub1_Pool2_Rep3_RPGC_mm10_scaled.bw
MINUTE_TSC3_EEDKO_H3K27me3_Pool2_Rep1_RPGC_mm10_scaled.bw
MINUTE_TSC3_EEDKO_H3K27me3_Pool2_Rep2_RPGC_mm10_scaled.bw
MINUTE_TSC3_EEDKO_H3K27me3_Pool2_Rep3_RPGC_mm10_scaled.bw
MINUTE_TSC3_EEDKO_H3K4me3_Pool2_Rep1_RPGC_mm10_scaled.bw
MINUTE_TSC3_EEDKO_H3K4me3_Pool2_Rep2_RPGC_mm10_scaled.bw
MINUTE_TSC3_EEDKO_H3K4me3_Pool2_Rep3_RPGC_mm10_scaled.bw
MINUTE_TSC3_EEDKO_Input_Pool2_Rep1_RPGC_mm10_unscaled.bw
MINUTE_TSC3_EEDKO_Input_Pool2_Rep2_RPGC_mm10_unscaled.bw
MINUTE_TSC3_EEDKO_Input_Pool2_Rep3_RPGC_mm10_unscaled.bw
MINUTE_ESC_WT_H2AK119ub1_Pool1_merged_mm10_scaled.bw
MINUTE_ESC_WT_H3K27me3_Pool1_merged_mm10_scaled.bw
MINUTE_ESC_WT_H3K4me3_Pool1_merged_mm10_scaled.bw
MINUTE_ESC_WT_Input_Pool1_merged_RPGC_mm10_unscaled.bw
MINUTE_TSC1_KDM2BKO_H2AK119ub1_Pool2_merged_mm10_scaled.bw
MINUTE_TSC1_KDM2BKO_H3K27me3_Pool2_merged_mm10_scaled.bw
MINUTE_TSC1_KDM2BKO_H3K4me3_Pool2_merged_mm10_scaled.bw
MINUTE_TSC1_KDM2BKO_Input_Pool2_merged_RPGC_mm10_unscaled.bw
MINUTE_TSC1_RNF2KO_H2AK119ub1_Pool2_merged_mm10_scaled.bw
MINUTE_TSC1_RNF2KO_H3K27me3_Pool2_merged_mm10_scaled.bw
MINUTE_TSC1_RNF2KO_H3K4me3_Pool2_merged_mm10_scaled.bw
MINUTE_TSC1_RNF2KO_Input_Pool2_merged_RPGC_mm10_unscaled.bw
MINUTE_TSC1_WT_EZH2i_5w_recovery_4w_H2AK119ub1_Pool2_merged_mm10_scaled.bw
MINUTE_TSC1_WT_EZH2i_5w_recovery_4w_H3K27me3_Pool2_merged_mm10_scaled.bw
MINUTE_TSC1_WT_EZH2i_5w_recovery_4w_H3K4me3_Pool2_merged_mm10_scaled.bw
MINUTE_TSC1_WT_EZH2i_5w_recovery_4w_Input_Pool2_merged_RPGC_mm10_unscaled.bw
MINUTE_TSC1_WT_H2AK119ub1_Pool1_merged_mm10_scaled.bw
MINUTE_TSC1_WT_H2AK119ub1_Pool2_merged_mm10_scaled.bw
MINUTE_TSC1_WT_H3K27me3_Pool1_merged_mm10_scaled.bw
MINUTE_TSC1_WT_H3K27me3_Pool2_merged_mm10_scaled.bw
MINUTE_TSC1_WT_H3K4me3_Pool1_merged_mm10_scaled.bw
MINUTE_TSC1_WT_H3K4me3_Pool2_merged_mm10_scaled.bw
MINUTE_TSC1_WT_Input_Pool1_merged_RPGC_mm10_unscaled.bw
MINUTE_TSC1_WT_Input_Pool2_merged_RPGC_mm10_unscaled.bw
MINUTE_TSC2_3BKO_H2AK119ub1_Pool1_merged_mm10_scaled.bw
MINUTE_TSC2_3BKO_H3K27me3_Pool1_merged_mm10_scaled.bw
MINUTE_TSC2_3BKO_H3K4me3_Pool1_merged_mm10_scaled.bw
MINUTE_TSC2_3BKO_Input_Pool1_merged_RPGC_mm10_unscaled.bw
MINUTE_TSC3_EEDKO_H2AK119ub1_Pool2_merged_mm10_scaled.bw
MINUTE_TSC3_EEDKO_H3K27me3_Pool2_merged_mm10_scaled.bw
MINUTE_TSC3_EEDKO_H3K4me3_Pool2_merged_mm10_scaled.bw
MINUTE_TSC3_EEDKO_Input_Pool2_merged_RPGC_mm10_unscaled.bw
ChIP-BS-seq_ESC_WT_H3K27me3_Input_Rep1_R1.fastq.gz ChIP-BS-seq_ESC_WT_H3K27me3_Input_Rep1_R2.fastq.gz
ChIP-BS-seq_ESC_WT_H3K27me3_Input_Rep2_R1.fastq.gz ChIP-BS-seq_ESC_WT_H3K27me3_Input_Rep2_R2.fastq.gz
ChIP-BS-seq_ESC_WT_H3K27me3_Rep1_R1.fastq.gz ChIP-BS-seq_ESC_WT_H3K27me3_Rep1_R2.fastq.gz
ChIP-BS-seq_ESC_WT_H3K27me3_Rep2_R1.fastq.gz ChIP-BS-seq_ESC_WT_H3K27me3_Rep2_R2.fastq.gz
ChIP-BS-seq_TSC1_WT_H3K27me3_Input_Rep1_R1.fastq.gz ChIP-BS-seq_TSC1_WT_H3K27me3_Input_Rep1_R2.fastq.gz
ChIP-BS-seq_TSC1_WT_H3K27me3_Input_Rep2_R1.fastq.gz ChIP-BS-seq_TSC1_WT_H3K27me3_Input_Rep2_R2.fastq.gz
ChIP-BS-seq_TSC1_WT_H3K27me3_Rep1_R1.fastq.gz ChIP-BS-seq_TSC1_WT_H3K27me3_Rep1_R2.fastq.gz
ChIP-BS-seq_TSC1_WT_H3K27me3_Rep2_R1.fastq.gz ChIP-BS-seq_TSC1_WT_H3K27me3_Rep2_R2.fastq.gz

ChIPseq_ESC_WT_EED_Input_Rep1_R1.fastq.gz ChIPseq_ESC_WT_EED_Input_Rep1_R2.fastq.gz
ChIPseq_ESC_WT_EED_Input_Rep2_R1.fastq.gz ChIPseq_ESC_WT_EED_Input_Rep2_R2.fastq.gz
ChIPseq_ESC_WT_EED_Rep1_R1.fastq.gz ChIPseq_ESC_WT_EED_Rep1_R2.fastq.gz
ChIPseq_ESC_WT_EED_Rep2_R1.fastq.gz ChIPseq_ESC_WT_EED_Rep2_R2.fastq.gz
ChIPseq_TSC1_WT_EED_Input_Rep1_R1.fastq.gz ChIPseq_TSC1_WT_EED_Input_Rep1_R2.fastq.gz
ChIPseq_TSC1_WT_EED_Input_Rep2_R1.fastq.gz ChIPseq_TSC1_WT_EED_Input_Rep2_R2.fastq.gz
ChIPseq_TSC1_WT_EED_Rep1_R1.fastq.gz ChIPseq_TSC1_WT_EED_Rep1_R2.fastq.gz
ChIPseq_TSC1_WT_EED_Rep2_R1.fastq.gz ChIPseq_TSC1_WT_EED_Rep2_R2.fastq.gz
ChIPseq_TSC1_WT_EZH2i_5w_4d_EED_Input_R1.fastq.gz ChIPseq_TSC1_WT_EZH2i_5w_4d_EED_Input_R2.fastq.gz
ChIPseq_TSC1_WT_EZH2i_5w_4d_EED_Rep1_R1.fastq.gz ChIPseq_TSC1_WT_EZH2i_5w_4d_EED_Rep1_R2.fastq.gz
ChIPseq_TSC1_WT_EZH2i_5w_4d_EED_Rep2_R1.fastq.gz ChIPseq_TSC1_WT_EZH2i_5w_4d_EED_Rep2_R2.fastq.gz
ChIP-BS-seq_ESC_WT_H3K27me3_Input_Rep1_RPGC_mm10.bw
ChIP-BS-seq_ESC_WT_H3K27me3_Input_Rep2_RPGC_mm10.bw
ChIP-BS-seq_ESC_WT_H3K27me3_Rep1_RPGC_mm10_input_subtracted.bw
ChIP-BS-seq_ESC_WT_H3K27me3_Rep2_RPGC_mm10_input_subtracted.bw
ChIP-BS-seq_ESC_WT_H3K27me3_merged_RPGC_mm10_input_subtracted.bw
ChIP-BS-seq_TSC1_WT_H3K27me3_Input_Rep1_RPGC_mm10.bw
ChIP-BS-seq_TSC1_WT_H3K27me3_Input_Rep2_RPGC_mm10.bw
ChIP-BS-seq_TSC1_WT_H3K27me3_Rep1_RPGC_mm10_input_subtracted.bw
ChIP-BS-seq_TSC1_WT_H3K27me3_Rep2_RPGC_mm10_input_subtracted.bw
ChIP-BS-seq_TSC1_WT_H3K27me3_merged_RPGC_mm10_input_subtracted.bw
ChIPseq_ESC_WT_EED_Input_Rep1_RPGC_mm10.bw
ChIPseq_ESC_WT_EED_Input_Rep2_RPGC_mm10.bw
ChIPseq_ESC_WT_EED_Rep1_RPGC_mm10_input_subtracted.bw
ChIPseq_ESC_WT_EED_Rep2_RPGC_mm10_input_subtracted.bw
ChIPseq_ESC_WT_EED_merged_RPGC_mm10_input_subtracted.bw
ChIPseq_TSC1_WT_EED_Input_Rep1_RPGC_mm10.bw
ChIPseq_TSC1_WT_EED_Input_Rep2_RPGC_mm10.bw
ChIPseq_TSC1_WT_EED_Rep1_RPGC_mm10_input_subtracted.bw
ChIPseq_TSC1_WT_EED_Rep2_RPGC_mm10_input_subtracted.bw
ChIPseq_TSC1_WT_EED_merged_RPGC_mm10_input_subtracted.bw
ChIPseq_TSC1_WT_EZH2i_5w_4d_EED_Input_RPGC_mm10.bw
ChIPseq_TSC1_WT_EZH2i_5w_4d_EED_Rep1_RPGC_mm10_input_subtracted.bw
ChIPseq_TSC1_WT_EZH2i_5w_4d_EED_Rep2_RPGC_mm10_input_subtracted.bw
ChIPseq_TSC1_WT_EZH2i_5w_4d_EED_merged_RPGC_mm10_input_subtracted.bw
ChIPseq_TSC1_WT_EED_merged_peaks_broad_mm10.bed
ChIPseq_TSC1_WT_EZH2i_5w_4d_EED_merged_peaks_broad_mm10.bed

Genome browser session (e.g. UCSC)

No longer applicable.

## Methodology

Replicates

MINUTE-ChIP experiments were performed in triplicates. ChIP-BS-seq and EED ChIP-seq experiments were performed in duplicates.

Sequencing depth

All MINUTE-ChIP, ChIP-BS-seq and ChIP-seq samples were sequenced using 100 bp paired-end reads.

Sample - Number of reads - Number of reads aligned
MINUTE_ESC_WT_H2AK119ub1_Pool1_merged 141845280 101941533
MINUTE_ESC_WT_H2AK119ub1_Pool1_Rep1 50065548 36684163
MINUTE_ESC_WT_H2AK119ub1_Pool1_Rep2 41595303 30350659
MINUTE_ESC_WT_H2AK119ub1_Pool1_Rep3 50184429 34906711
MINUTE_TSC2_3BKO_H2AK119ub1_Pool1_merged 243588532 168567006
MINUTE_TSC2_3BKO_H2AK119ub1_Pool1_Rep1 80878090 55134033
MINUTE_TSC2_3BKO_H2AK119ub1_Pool1_Rep2 113100219 78952552
MINUTE_TSC2_3BKO_H2AK119ub1_Pool1_Rep3 49610223 34480421
MINUTE_TSC1_WT_H2AK119ub1_Pool1_merged 211698526 138859385
MINUTE_TSC1_WT_H2AK119ub1_Pool1_Rep1 78924631 53817493
MINUTE_TSC1_WT_H2AK119ub1_Pool1_Rep2 52123133 32688503
MINUTE_TSC1_WT_H2AK119ub1_Pool1_Rep3 80650762 52353389
MINUTE_ESC_WT_Input_Pool1_merged 343753429 151525584
MINUTE_ESC_WT_Input_Pool1_Rep1 102646276 54188917
MINUTE_ESC_WT_Input_Pool1_Rep2 118151325 43566752
MINUTE_ESC_WT_Input_Pool1_Rep3 122955828 53769915
MINUTE_TSC2_3BKO_Input_Pool1_merged 355408705 172183271
MINUTE_TSC2_3BKO_Input_Pool1_Rep1 99916131 53923869
MINUTE_TSC2_3BKO_Input_Pool1_Rep2 144545605 81663723
MINUTE_TSC2_3BKO_Input_Pool1_Rep3 110946969 36595679
MINUTE_TSC1_WT_Input_Pool1_merged 596699276 208711190
MINUTE_TSC1_WT_Input_Pool1_Rep1 273763826 88273170
MINUTE_TSC1_WT_Input_Pool1_Rep2 145001817 46244776
MINUTE_TSC1_WT_Input_Pool1_Rep3 177933633 74193244
MINUTE_ESC_WT_H3K27me3_Pool1_merged 32719139 19339355
MINUTE_ESC_WT_H3K27me3_Pool1_Rep1 11262487 7003712
MINUTE_ESC_WT_H3K27me3_Pool1_Rep2 8720460 5328786
MINUTE_ESC_WT_H3K27me3_Pool1_Rep3 12736192 7006857

MINUTE_TSC2_3BKO_H3K27me3_Pool1_merged 233603994 157500268
MINUTE_TSC2_3BKO_H3K27me3_Pool1_Rep1 76548171 50633401
MINUTE_TSC2_3BKO_H3K27me3_Pool1_Rep2 105486623 71866746
MINUTE_TSC2_3BKO_H3K27me3_Pool1_Rep3 51569200 35000121
MINUTE_TSC1_WT_H3K27me3_Pool1_merged 238672876 158569921
MINUTE_TSC1_WT_H3K27me3_Pool1_Rep1 87076293 60105343
MINUTE_TSC1_WT_H3K27me3_Pool1_Rep2 63859004 40560155
MINUTE_TSC1_WT_H3K27me3_Pool1_Rep3 87737579 57904423
MINUTE_ESC_WT_H3K4me3_Pool1_merged 186788627 148862331
MINUTE_ESC_WT_H3K4me3_Pool1_Rep1 67127140 55120682
MINUTE_ESC_WT_H3K4me3_Pool1_Rep2 51277688 39859044
MINUTE_ESC_WT_H3K4me3_Pool1_Rep3 68383799 53882605
MINUTE_TSC2_3BKO_H3K4me3_Pool1_merged 215014259 156808573
MINUTE_TSC2_3BKO_H3K4me3_Pool1_Rep1 68458242 50269827
MINUTE_TSC2_3BKO_H3K4me3_Pool1_Rep2 96640512 71477965
MINUTE_TSC2_3BKO_H3K4me3_Pool1_Rep3 49915505 35060781
MINUTE_TSC1_WT_H3K4me3_Pool1_merged 275362302 191912576
MINUTE_TSC1_WT_H3K4me3_Pool1_Rep1 111144193 79228100
MINUTE_TSC1_WT_H3K4me3_Pool1_Rep2 68590298 44988541
MINUTE_TSC1_WT_H3K4me3_Pool1_Rep3 95627811 67695935
MINUTE_TSC1_WT_EZH2i_5w_recovery_4w_H2AK119ub1_Pool2_merged 168298808 90733792
MINUTE_TSC1_WT_EZH2i_5w_recovery_4w_H2AK119ub1_Pool2_Rep1 57427876 31694730
MINUTE_TSC1_WT_EZH2i_5w_recovery_4w_H2AK119ub1_Pool2_Rep2 59219965 30329923
MINUTE_TSC1_WT_EZH2i_5w_recovery_4w_H2AK119ub1_Pool2_Rep3 51650967 28709139
MINUTE_TSC1_WT_H2AK119ub1_Pool2_merged 213901307 106497392
MINUTE_TSC1_WT_H2AK119ub1_Pool2_Rep1 65674827 34936402
MINUTE_TSC1_WT_H2AK119ub1_Pool2_Rep2 40497725 20878610
MINUTE_TSC1_WT_H2AK119ub1_Pool2_Rep3 107728755 50682380
MINUTE_TSC3_EEDKO_H2AK119ub1_Pool2_merged 218043557 126532156
MINUTE_TSC3_EEDKO_H2AK119ub1_Pool2_Rep1 73153206 44177404
MINUTE_TSC3_EEDKO_H2AK119ub1_Pool2_Rep2 60947244 34445412
MINUTE_TSC3_EEDKO_H2AK119ub1_Pool2_Rep3 83943107 47909340
MINUTE_TSC1_KDM2BKO_H2AK119ub1_Pool2_merged 153309045 89700547
MINUTE_TSC1_KDM2BKO_H2AK119ub1_Pool2_Rep1 56510675 34377032
MINUTE_TSC1_KDM2BKO_H2AK119ub1_Pool2_Rep2 42440202 23235706
MINUTE_TSC1_KDM2BKO_H2AK119ub1_Pool2_Rep3 54358168 32087809
MINUTE_TSC1_RNF2KO_H2AK119ub1_Pool2_merged 75530458 42988929
MINUTE_TSC1_RNF2KO_H2AK119ub1_Pool2_Rep1 19802076 10471992
MINUTE_TSC1_RNF2KO_H2AK119ub1_Pool2_Rep2 29662427 17982267
MINUTE_TSC1_RNF2KO_H2AK119ub1_Pool2_Rep3 26065955 14534670
MINUTE_TSC1_WT_Input_Pool2_merged 328722233 132495967
MINUTE_TSC1_WT_Input_Pool2_Rep1 100487642 46157234
MINUTE_TSC1_WT_Input_Pool2_Rep2 90249694 26241606
MINUTE_TSC1_WT_Input_Pool2_Rep3 137984897 60097127
MINUTE_TSC3_EEDKO_Input_Pool2_merged 296078609 119642065
MINUTE_TSC3_EEDKO_Input_Pool2_Rep1 87229727 41504715
MINUTE_TSC3_EEDKO_Input_Pool2_Rep2 99055499 31977906
MINUTE_TSC3_EEDKO_Input_Pool2_Rep3 109793383 46159444
MINUTE_TSC1_WT_EZH2i_5w_recovery_4w_Input_Pool2_merged 364789499 139552924
MINUTE_TSC1_WT_EZH2i_5w_recovery_4w_Input_Pool2_Rep1 124130256 47985369
MINUTE_TSC1_WT_EZH2i_5w_recovery_4w_Input_Pool2_Rep2 128732348 49841001
MINUTE_TSC1_WT_EZH2i_5w_recovery_4w_Input_Pool2_Rep3 111926895 41726554
MINUTE_TSC1_KDM2BKO_Input_Pool2_merged 390301863 168868264
MINUTE_TSC1_KDM2BKO_Input_Pool2_Rep1 160768318 67200838
MINUTE_TSC1_KDM2BKO_Input_Pool2_Rep2 108897239 42000159
MINUTE_TSC1_KDM2BKO_Input_Pool2_Rep3 120636306 59667267
MINUTE_TSC1_RNF2KO_Input_Pool2_merged 272464823 121777220
MINUTE_TSC1_RNF2KO_Input_Pool2_Rep1 62685809 30861253
MINUTE_TSC1_RNF2KO_Input_Pool2_Rep2 92580072 49249174
MINUTE_TSC1_RNF2KO_Input_Pool2_Rep3 117198942 41666793
MINUTE_TSC1_WT_EZH2i_5w_recovery_4w_H3K27me3_Pool2_merged 236356961 130738765
MINUTE_TSC1_WT_EZH2i_5w_recovery_4w_H3K27me3_Pool2_Rep1 73809591 41069794
MINUTE_TSC1_WT_EZH2i_5w_recovery_4w_H3K27me3_Pool2_Rep2 88303582 46927267
MINUTE_TSC1_WT_EZH2i_5w_recovery_4w_H3K27me3_Pool2_Rep3 74243788 42741704
MINUTE_TSC1_WT_H3K27me3_Pool2_merged 245067144 123141814
MINUTE_TSC1_WT_H3K27me3_Pool2_Rep1 73096464 39140708
MINUTE_TSC1_WT_H3K27me3_Pool2_Rep2 48309268 25453463
MINUTE_TSC1_WT_H3K27me3_Pool2_Rep3 123661412 58547643
MINUTE_TSC3_EEDKO_H3K27me3_Pool2_merged 34483059 14090270
MINUTE_TSC3_EEDKO_H3K27me3_Pool2_Rep1 10833672 4652161
MINUTE_TSC3_EEDKO_H3K27me3_Pool2_Rep2 8603252 3401434
MINUTE_TSC3_EEDKO_H3K27me3_Pool2_Rep3 15046135 6036675
MINUTE_TSC1_KDM2BKO_H3K27me3_Pool2_merged 165233328 98350986
MINUTE_TSC1_KDM2BKO_H3K27me3_Pool2_Rep1 59795267 36638260
MINUTE_TSC1_KDM2BKO_H3K27me3_Pool2_Rep2 46351310 25983293
MINUTE_TSC1_KDM2BKO_H3K27me3_Pool2_Rep3 59086751 35729433

MINUTE_TSC1_RNF2KO_H3K27me3_Pool2_merged 128076569 77314618
MINUTE_TSC1_RNF2KO_H3K27me3_Pool2_Rep1 30589049 17320342
MINUTE_TSC1_RNF2KO_H3K27me3_Pool2_Rep2 50906169 32635015
MINUTE_TSC1_RNF2KO_H3K27me3_Pool2_Rep3 46581351 27359261
MINUTE_TSC1_WT_EZH2i_5w_recovery_4w_H3K4me3_Pool2_merged 226774674 128311244
MINUTE_TSC1_WT_EZH2i_5w_recovery_4w_H3K4me3_Pool2_Rep1 78229362 43866195
MINUTE_TSC1_WT_EZH2i_5w_recovery_4w_H3K4me3_Pool2_Rep2 77863358 43801880
MINUTE_TSC1_WT_EZH2i_5w_recovery_4w_H3K4me3_Pool2_Rep3 70681954 40643169
MINUTE_TSC1_WT_H3K4me3_Pool2_merged 266836087 139662202
MINUTE_TSC1_WT_H3K4me3_Pool2_Rep1 81635204 45806411
MINUTE_TSC1_WT_H3K4me3_Pool2_Rep2 52228585 27050950
MINUTE_TSC1_WT_H3K4me3_Pool2_Rep3 132972298 66804841
MINUTE_TSC3_EEDKO_H3K4me3_Pool2_merged 175443574 100432930
MINUTE_TSC3_EEDKO_H3K4me3_Pool2_Rep1 59024385 35500868
MINUTE_TSC3_EEDKO_H3K4me3_Pool2_Rep2 47322664 25918081
MINUTE_TSC3_EEDKO_H3K4me3_Pool2_Rep3 69096525 39013981
MINUTE_TSC1_KDM2BKO_H3K4me3_Pool2_merged 255355318 160441580
MINUTE_TSC1_KDM2BKO_H3K4me3_Pool2_Rep1 99107491 63055348
MINUTE_TSC1_KDM2BKO_H3K4me3_Pool2_Rep2 66902809 39917029
MINUTE_TSC1_KDM2BKO_H3K4me3_Pool2_Rep3 89345018 57469203
MINUTE_TSC1_RNF2KO_H3K4me3_Pool2_merged 149092308 99126770
MINUTE_TSC1_RNF2KO_H3K4me3_Pool2_Rep1 37149959 23977642
MINUTE_TSC1_RNF2KO_H3K4me3_Pool2_Rep2 58733615 40704248
MINUTE_TSC1_RNF2KO_H3K4me3_Pool2_Rep3 53208734 34444880
ChIP-BS-seq_ESC_WT_H3K27me3_Rep1 164101626 140738559
ChIP-BS-seq_ESC_WT_H3K27me3_Input_Rep1 203837836 171835894
ChIP-BS-seq_ESC_WT_H3K27me3_Rep2 130882570 111365421
ChIP-BS-seq_ESC_WT_H3K27me3_Input_Rep2 150804558 126779101
ChIP-BS-seq_TSC1_WT_H3K27me3_Rep1 151500914 130089160
ChIP-BS-seq_TSC1_WT_H3K27me3_Input_Rep1 135742638 108544707
ChIP-BS-seq_TSC1_WT_H3K27me3_Rep2 133212844 116169501
ChIP-BS-seq_TSC1_WT_H3K27me3_Input_Rep2 138307810 113594936
ChIPseq_ESC_WT_EED_Rep1 110872064 109275287
ChIPseq_ESC_WT_EED_Input_Rep1 176371316 173280612
ChIPseq_ESC_WT_EED_Rep2 95414054 93546165
ChIPseq_ESC_WT_EED_Input_Rep2 113403308 112652634
ChIPseq_TSC1_WT_EED_Rep1 138446050 136848653
ChIPseq_TSC1_WT_EED_Input_Rep1 131513134 129550215
ChIPseq_TSC1_WT_EED_Rep2 106175636 104559800
ChIPseq_TSC1_WT_EED_Input_Rep2 101350538 100553303
ChIPseq_TSC1_WT_EZH2i_5w_4d_EED_Rep1 134135868 132238906
ChIPseq_TSC1_WT_EZH2i_5w_4d_EED_Rep2 136643690 134987043
ChIPseq_TSC1_WT_EZH2i_5w_4d_EED_Input 94794548 94117771

Antibodies

MINUTE-ChIP:
Rabbit anti-H3K4me3: Millipore, #04-745, clone: MC315
Rabbit anti-H3K27me3: Cell Signaling, #9733, clone: C36B11
Rabbit anti-H2AK119ub: Cell Signaling, #8240, clone: D27C4

ChIP-BS-seq:
Rabbit anti-H3K27me3: Thermo Fisher, MA5-11198, lot: WH3366172

ChIP-seq:
Rabbit anti-EED: ab240650, EPR23043-5, lot: GR3427609-2

Peak calling parameters

Peaks for EED ChIPs were called using MACS2 'callpeak' (version 2.1.2; parameters --bdg --SPMR --broad) based on merged replicates using the input samples as control samples and only peaks with a q-value < 0.01 were considered for downstream analyses. No peak calling was performed for MINUTE-ChIP data generated within this study. Instead the scaled RPGC values were used to calculate the average intensity across CpG islands or one kb tiles. The tracks were generated as described below (Software).

Data quality

FastQC was run on all FASTQ files to assess general sequencing quality. Picard was used to determine insert size distribution, duplication rate, estimated library size. Mapping stats were generated from BAM files using samtools idxstats and flagstat commands. Final reports with all the statistics generated throughout the pipeline execution are gathered with MultiQC.
For EED ChIP-seq samples, peaks were called on the merged replicates. For WT TSCs 19,775 broad peaks were called with an FDR < 0.01. For WT TSCs treated with EZH2i 7,553 broad peaks were called with an FDR < 0.01.

Software

MINUTE-ChIP processing:
MINUTE-ChIP multiplexed FASTQ files were processed using minute, a workflow implemented in Snakemake. In order to ensure reproducibility, a conda environment was set up. Source code and documentation are fully available on GitHub: https://github.com/NBISweden/minute. Main steps performed are described below.
Adaptor removal: Read pairs matching parts of the adaptor sequence (SBS3 or T7 promoter) in either read1 or read2 were removed using cutadapt v3.2.

Demultiplexing and deduplication: Reads were demultiplexed using cutadapt v3.2 allowing only one mismatch per barcode and written into sample-specific FASTQ files used for subsequent mapping.
Mapping: Sample-specific paired FASTQ files were mapped to the reference mm10 using bowtie2 v2.3.5.1 with --fast and --reorder parameter. Alignments were processed into sorted BAM files and replicates were pooled using samtools v1.10.
Deduplication: Duplicate reads are marked using UMI-sensitive deduplication tool je-suite (v2.0.RC) (https://github.com/gbcs-embl/Je/). Read pairs are marked as duplicates if their read1 (first-in-pair) sequences have the same UMI (allowing for 1 mismatch) and map to the same location in the genome. Blacklisted regions as downloaded from ENCODE were then removed from BAM files using bedtools v2.30.
Generation of coverage tracks and quantitative scaling: Input coverage tracks with 1 bp resolution in bigWig format were generated from BAM files using deepTools v3.5.0 bamCoverage and scaled to a reads-per-genome-coverage of one (1xRPGC, also referred to as '1x normalization') using the mm10 effective genome size. ChIP coverage tracks were generated from BAM files using deepTools (v3.5.0) bamCoverage. Quantitative scaling of the ChIP-Seq tracks amongst conditions within each pool was based on their Input-Normalized Mapped Read Count (INRC). INRC was calculated by dividing the number of unique reference-mapped reads by the respective number of Input reads: #mapped[ChIP] / #mapped[Input]. This essentially corrects for an uneven representation of barcodes in the Input. It has been previously shown that INRCs are proportional to the amount of epitope present in each condition. Reference condition (TSC WT) was scaled to 1x coverage (also termed Reads per Genome Coverage, RPGC). All other conditions within the same pool were scaled relative to the reference using the ratio of INRCs multiplied by the scaling factor determined for 1x normalization of the reference: ( #mapped[ChIP] / #mapped[Input] ) / ( #mapped[ChIP_Reference] / #mapped[Input_Reference] ) * scaling factor.

ChIP-BS-seq processing:
Raw reads of ESC and TSC H3K27me3 ChIP-BS-seq samples as well as their respective input samples were subjected to adapter and quality trimming with cutadapt (version 2.4; parameters: --quality-cutoff 20 --overlap 5 --minimum-length 25 --adapter AGATCGGAAGAGC -A AGATCGGAAGAGC). Reads were aligned to the mouse genome (mm10) using BSMAP (version 2.90; parameters: -v 0.1 -s 16 -q 20 -w 100 -S 1 -u -R). A sorted BAM file was obtained and indexed using samtools with the 'sort' and 'index' commands (version 1.10). Duplicate reads were identified and removed using GATK (version 4.1.4.1) 'MarkDuplicates' and default parameters. After careful inspection and validation of high correlation, replicates of treatment and input samples were merged respectively using samtools 'merge'. Methylation rates were called using mcall from the MOABS package (version 1.3.2; default parameters). All analyses were restricted to autosomes and only CpGs covered by at least 10 and at most 150 reads were considered for downstream analyses. Genome-wide coverage tracks for single and merged replicates normalized by library size were computed using deepTools bamCoverage (parameters: --normalizeUsing RPGC --extendReads --smoothLength 300). Coverage tracks were subtracted by the respective input using deeptools 'bigwigCompare'.

ChIP-seq processing:
Raw reads of ESC and TSC EED and publicly available ESC H3K27me3 ChIP-seq samples as well as their respective input samples were subjected to adapter and quality trimming with cutadapt (version 2.4; parameters: --quality-cutoff 20 --overlap 5 --minimum-length 25 --adapter AGATCGGAAGAGC -A AGATCGGAAGAGC). Reads were aligned to the mouse genome (mm10) using BWA with the 'mem' command (version 0.7.17, default parameters)61. A sorted BAM file was obtained and indexed using samtools with the 'sort' and 'index' commands (version 1.10)66. Duplicate reads were identified and removed using GATK (version 4.1.4.1) 'MarkDuplicates' and default parameters. After careful inspection and validation of high correlation, replicates of treatment and input samples were merged respectively using samtools 'merge'. Domains for public H3K27me3 ESC samples were called for each sample with its respective input using peakranger 'bcp' (version 1.18) 67. Only regions called a domain in at least two of the samples were considered for the final selection and merged using bedtools 'mergeBed' (parameters: -d 50). Retained regions smaller than 100 bp were removed from the set. Peaks for EED ChIPs were called using MACS2 'callpeak' (version 2.1.2; parameters --bdg --SPMR --broad) based on merged replicates using the input samples as control samples and only peaks with a q-value < 0.01 were considered for downstream analyses. Genome-wide coverage tracks for single and merged replicates normalized by library size were computed using deepTools bamCoverage (parameters: --normalizeUsing RPGC --extendReads --smoothLength 300). Coverage tracks were subtracted by the respective input using deeptools 'bigwigCompare'.

# Flow Cytometry

## Plots

Confirm that:

☒ The axis labels state the marker and fluorochrome used (e.g. CD4-FITC).

☒ The axis scales are clearly visible. Include numbers along axes only for bottom left plot of group (a 'group' is an analysis of identical markers).

☒ All plots are contour plots with outliers or pseudocolor plots.

☒ A numerical value for number of cells or percentage (with statistics) is provided.

## Methodology

| | |
|---|---|
| Sample preparation | Cells were detached using Trypsin-EDTA 0.05 % for 10 minutes. Subsequently, trypsinization was stopped by addition of ESC/TSC medium containing FBS and cells were dissociated to generate a single cell suspension. Cells were spun down, washed once with PBS and passed through a FACS tube with cell strainer just before the sort with the flow cytometer |
| Instrument | BD FACS Aria II and BD FACS Fusion |
| Software | FACS Diva (BD Biosciences) for collection and FlowJo (v1.07) for analysis |

Cell population abundance

The overall cell population was calculated using forward and side-scatter patterns. The abundance of cells in a population is represented as the normalized mode.

Gating strategy

Gating for negative and positive population was determined with untreated or isotype controls.

☒ Tick this box to confirm that a figure exemplifying the gating strategy is provided in the Supplementary Information.
