# Supplementary figures and images for: Dynamic antagonism between key repressive pathways maintains the placental epigenome
Source: Nat Cell Biol. 2023 Apr 6;25(4):579–91. doi: 10.1038/s41556-023-01114-y (PMC10104784; doi:10.1038/s41556-023-01114-y)

Source data for Figure 5c:

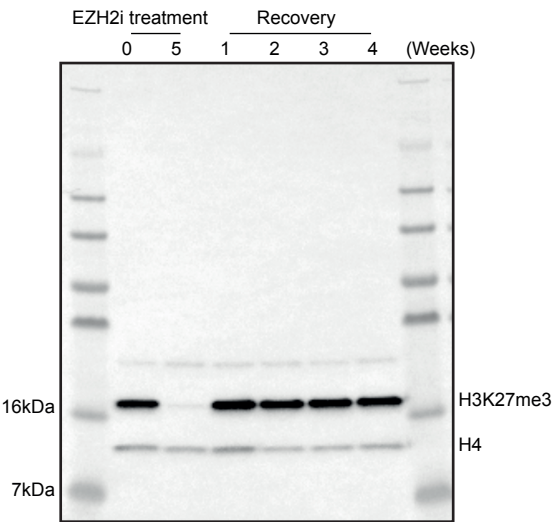

Source data for Figure 5d:

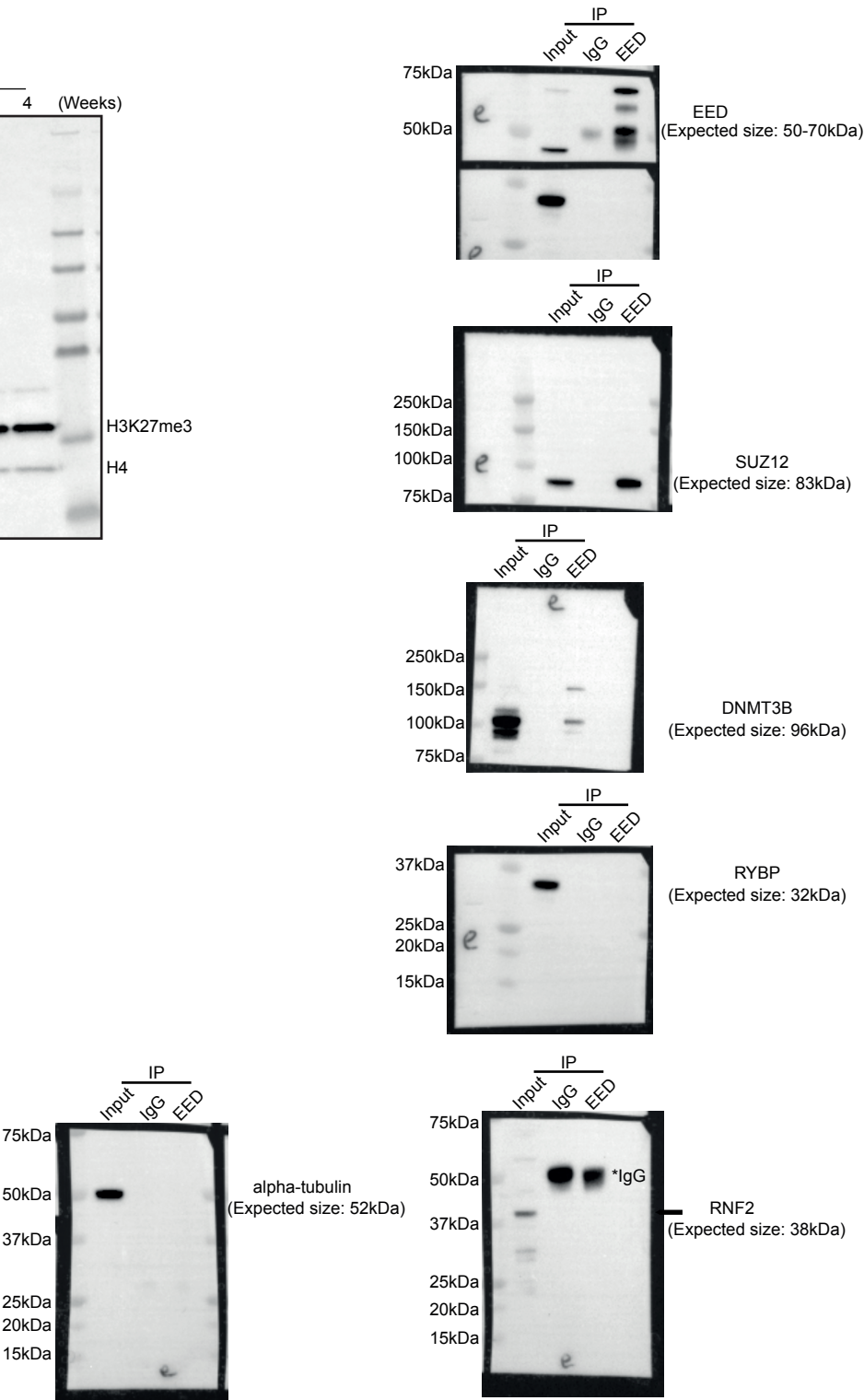

Supplement: Supplementary file 5 — Unprocessed western blots. [file 41556_2023_1114_MOESM5_ESM.pdf]

Source data for ED 1d:

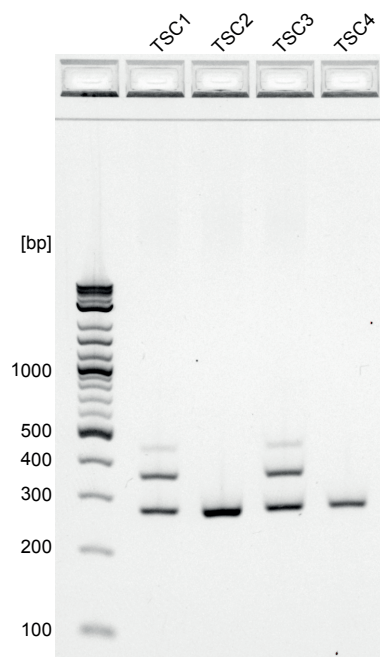

Supplement: Supplementary file 6 — Unprocessed gels. [file 41556_2023_1114_MOESM6_ESM.pdf]

Source data for ED Fig. 3e:

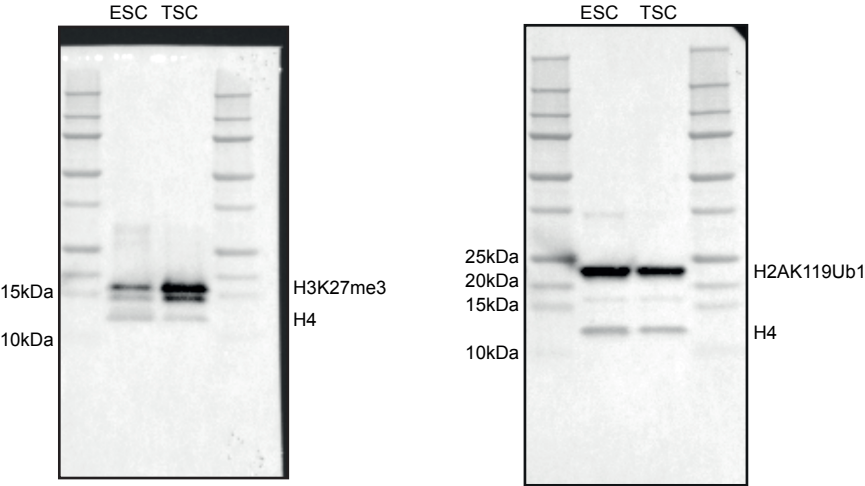

Supplement: Supplementary file 7 — Unprocessed western blots. [file 41556_2023_1114_MOESM7_ESM.pdf]

Source data for ED Fig. 5f:

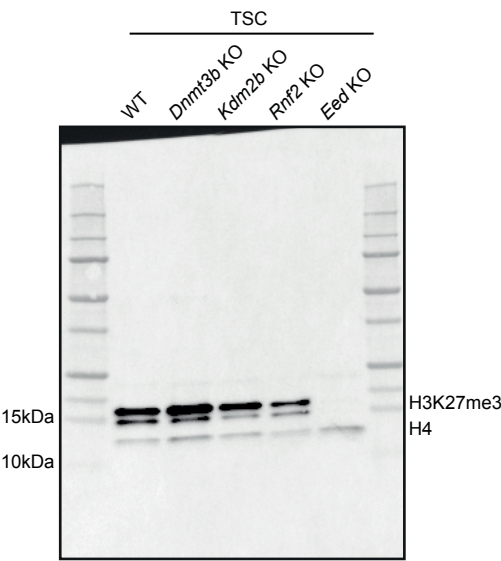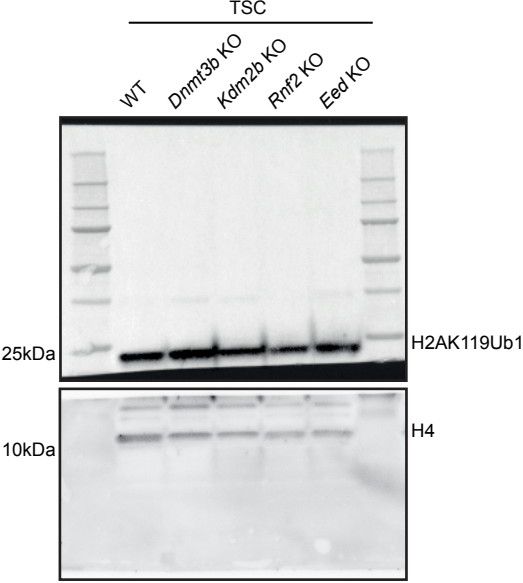

Supplement: Supplementary file 8 — Unprocessed western blots. [file 41556_2023_1114_MOESM8_ESM.pdf]

Source data for ED Fig. 8c:

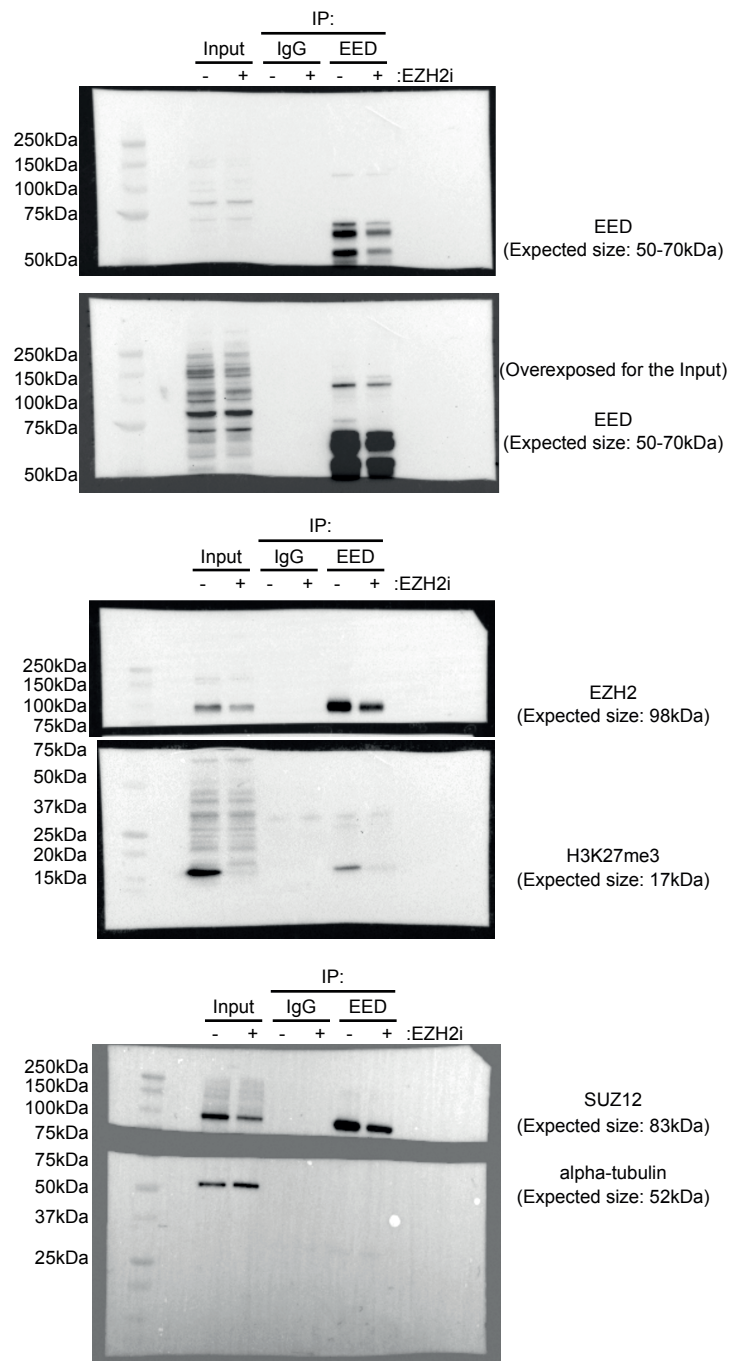

Source data for ED Fig. 8g:

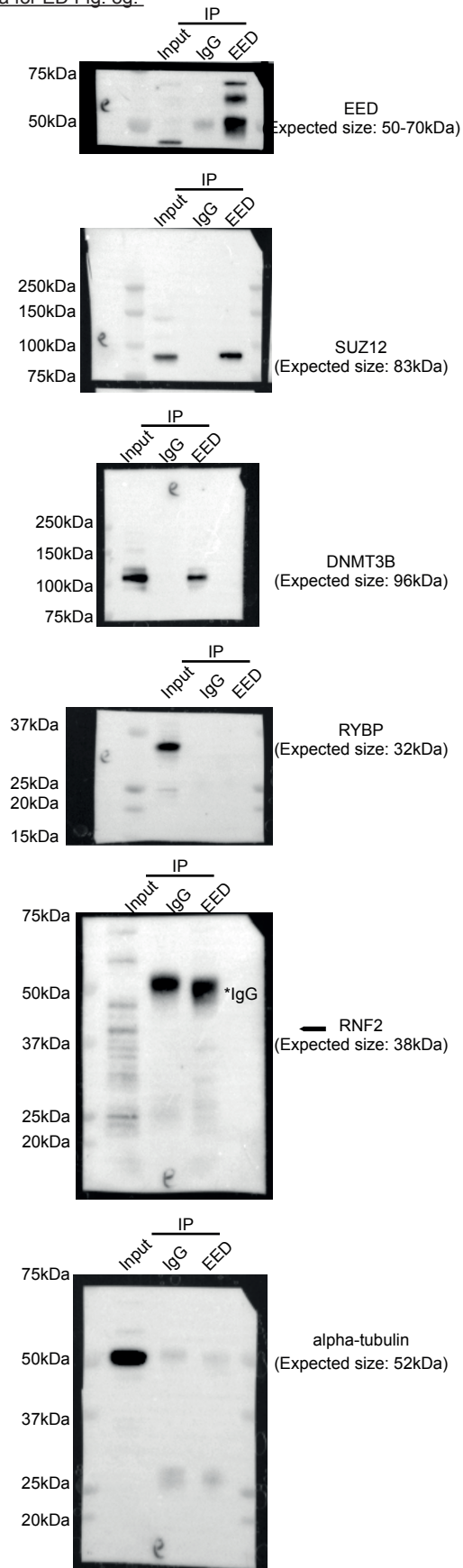

Supplement: Supplementary file 9 — Unprocessed western blots. [file 41556_2023_1114_MOESM9_ESM.pdf]
